# Supplementary material for: Neogene paleogeography provides context for understanding the origin and spatial distribution of cryptic diversity in a widespread Balkan freshwater amphipod
Source: PeerJ. 2017 Feb 28;5:e3016. doi: 10.7717/peerj.3016 (PMC5333542; doi:10.7717/peerj.3016)
Supplement: Table S1 — All analysed individuals with BOLD process ID, sample ID, Barcode Index numbers (BIN) and their COI haplotype membership, primers pairs used for amplification. [file peerj-05-3016-s001.docx]

| BOLD | | | COI | |
| --- | --- | --- | --- | --- |
| Process ID | Sample ID | BIN | haplotype | primers pair |
| GRBAL040-16 | 10-GR05-1 | BOLD:ACZ6711 | C38 | UCOIF+UCOIR |
| GRBAL042-16 | 10-GR05-4 | BOLD:ACZ6711 | C42 | UCOIF+UCOIR |
| GRBAL041-16 | 10-GR05-6 | BOLD:ACZ6711 | C39 | UCOIF+UCOIR |
| GRBAL046-16 | 11-LIB-AL35-2 | BOLD:ACH5401 | C43 | UCOIF+UCOIR |
| GRBAL043-16 | 11-LIB-AL35-3 | BOLD:ADD3859 | C22 | UCOIF+UCOIR |
| GRBAL044-16 | 11-LIB-AL35-4 | BOLD:ACH5401 | C36 | UCOIF+UCOIR |
| GRBAL045-16 | 11-LIB-AL35-6 | BOLD:ACH5401 | C37 | UCOIF+UCOIR |
| GRBAL047-16 | 12-ORI-AL33-1 | BOLD:ADD3858 | C59 | UCOIF+UCOIR |
| GRBAL050-16 | 12-ORI-AL33-2 | BOLD:ADD3858 | C62 | COIGrF+COIGrR2 |
| GRBAL048-16 | 12-ORI-AL33-4 | BOLD:ADD3858 | C60 | UCOIF+UCOIR |
| GRBAL049-16 | 12-ORI-AL33-5 | BOLD:ADD3858 | C61 | UCOIF+UCOIR |
| GRBAL052-16 | 13-AL45-1 | BOLD:ADD2307 | C49 | UCOIF+UCOIR |
| GRBAL058-16 | 13-AL45-13 | BOLD:ADD2307 | C51 | UCOIF+UCOIR |
| GRBAL056-16 | 13-AL45-15 | BOLD:ADD3589 | C50 | UCOIF+UCOIR |
| GRBAL055-16 | 13-AL45-16 | BOLD:ADD2307 | C49 | UCOIF+UCOIR |
| GRBAL060-16 | 13-AL45-17 | BOLD:ADD3863 | C64 | UCOIF+UCOIR |
| GRBAL061-16 | 13-AL45-18 | BOLD:ADD3863 | C64 | UCOIF+UCOIR |
| GRBAL053-16 | 13-AL45-2 | BOLD:ADD2307 | C49 | UCOIF+UCOIR |
| GRBAL059-16 | 13-AL45-20 | BOLD:ADD3863 | C63 | UCOIF+UCOIR |
| GRBAL062-16 | 13-AL45-22 | BOLD:ADD3863 | C64 | UCOIF+UCOIR |
| GRBAL051-16 | 13-AL45-23 | BOLD:ADD0784 | C48 | UCOIF+UCOIR |
| GRBAL054-16 | 13-AL45-3 | BOLD:ADD2307 | C49 | UCOIF+UCOIR |
| GRBAL057-16 | 13-AL45-4 | BOLD:ADD2307 | C51 | UCOIF+UCOIR |
| GRBAL068-16 | 14-GR14-10 | BOLD:ADD3862 | C88 | LCO1490+HCO2198 |
| GRBAL072-16 | 14-GR14-11 | BOLD:ADD3862 | C90 | LCO1490+HCO2198 |
| GRBAL066-16 | 14-GR14-12 | BOLD:ADD3862 | C86 | LCO1490+HCO2198 |
| GRBAL070-16 | 14-GR14-2 | BOLD:ADD3862 | C90 | LCO1490+HCO2198 |
| GRBAL063-16 | 14-GR14-3 | BOLD:ADD3862 | C84 | LCO1490+HCO2198 |
| GRBAL069-16 | 14-GR14-4 | BOLD:ADD3862 | C89 | LCO1490+HCO2198 |
| GRBAL064-16 | 14-GR14-5 | BOLD:ADD3862 | C85 | LCO1490+HCO2198 |
| GRBAL065-16 | 14-GR14-7 | BOLD:ADD3862 | C86 | LCO1490+HCO2198 |
| GRBAL071-16 | 14-GR14-8 | BOLD:ADD3862 | C90 | LCO1490+HCO2198 |
| GRBAL067-16 | 14-GR14-9 | BOLD:ADD3862 | C87 | LCO1490+HCO2198 |
| GRBAL076-16 | 15-GR19-1 | BOLD:ADD2747 | C68 | LCO1490+HCO2198 |
| GRBAL074-16 | 15-GR19-10 | BOLD:ADD2747 | C66 | LCO1490+HCO2198 |
| GRBAL083-16 | 15-GR19-11 | BOLD:ADD2747 | C71 | UCOIF+UCOIR |
| GRBAL084-16 | 15-GR19-12 | BOLD:ADD2747 | C71 | LCO1490+HCO2198 |
| GRBAL077-16 | 15-GR19-2 | BOLD:ADD2747 | C69 | LCO1490+HCO2198 |
| GRBAL078-16 | 15-GR19-3 | BOLD:ADD2747 | C70 | LCO1490+HCO2198 |
| GRBAL079-16 | 15-GR19-4 | BOLD:ADD2747 | C71 | LCO1490+HCO2198 |
| GRBAL080-16 | 15-GR19-5 | BOLD:ADD2747 | C71 | LCO1490+HCO2198 |
| GRBAL081-16 | 15-GR19-6 | BOLD:ADD2747 | C70 | LCO1490+HCO2198 |
| GRBAL075-16 | 15-GR19-8 | BOLD:ADD2747 | C67 | UCOIF+UCOIR |
| GRBAL082-16 | 15-GR19-9 | BOLD:ADD2747 | C71 | LCO1490+HCO2198 |
| GRBAL073-16 | 15-GR19-GRLLA1 | BOLD:ADD2747 | C65 | LCO1490+HCO2198 |
| GRBAL085-16 | 16-GR02- MAC02-4 | BOLD:ADD0638 | C7 | UCOIF+UCOIR |
| GRBAL087-16 | 16-GR02 MAC02-6 | BOLD:ADD0638 | C7 | UCOIF+UCOIR |
| GRBAL092-16 | 16-GR02-MAC02-1 | BOLD:ADD3389 | C21 | LCO1490+HCO2198 |
| GRBAL090-16 | 16-GR02-MAC02-10 | BOLD:ADD0638 | C7 | UCOIF+UCOIR |
| GRBAL091-16 | 16-GR02-MAC02-11 | BOLD:ADD0638 | C7 | UCOIF+UCOIR |
| GRBAL093-16 | 16-GR02-MAC02-3 | BOLD:ADD3389 | C21 | LCO1490+HCO2198 |
| GRBAL086-16 | 16-GR02-MAC02-5 | BOLD:ADD0638 | C7 | UCOIF+UCOIR |
| GRBAL088-16 | 16-GR02-MAC02-8 | BOLD:ADD0638 | C7 | UCOIF+UCOIR |
| GRBAL089-16 | 16-GR02-MAC02-9 | BOLD:ADD0638 | C7 | UCOIF+UCOIR |
| GRBAL099-16 | 17-GR09-11 | BOLD:ADD1232 | C1 | UCOIF+UCOIR |
| GRBAL101-16 | 17-GR09-13 | BOLD:ADD1232 | C2 | UCOIF+UCOIR |
| GRBAL094-16 | 17-GR09-2 | BOLD:ADD1232 | C1 | LCO1490+HCO2198 |
| GRBAL095-16 | 17-GR09-3 | BOLD:ADD1232 | C1 | UCOIF+UCOIR |
| GRBAL096-16 | 17-GR09-4 | BOLD:ADD1232 | C1 | UCOIF+UCOIR |
| GRBAL100-16 | 17-GR09-6 | BOLD:ADD1232 | C2 | LCO1490+HCO2198 |
| GRBAL097-16 | 17-GR09-7 | BOLD:ADD1232 | C1 | UCOIF+UCOIR |
| GRBAL102-16 | 17-GR09-8 | BOLD:ADD1232 | C9 | UCOIF+UCOIR |
| GRBAL098-16 | 17-GR09-9 | BOLD:ADD1232 | C1 | UCOIF+UCOIR |
| GRBAL107-16 | 18-GR10-1 | BOLD:ADD1232 | C8 | UCOIF+UCOIR |
| GRBAL109-16 | 18-GR10-11 | BOLD:ADD1232 | C18 | UCOIF+UCOIR |
| GRBAL104-16 | 18-GR10-2 | BOLD:ADD1232 | C4 | UCOIF+UCOIR |
| GRBAL105-16 | 18-GR10-3 | BOLD:ADD1232 | C4 | UCOIF+UCOIR |
| GRBAL111-16 | 18-GR10-4 | BOLD:ADD1232 | C19 | UCOIF+UCOIR |
| GRBAL103-16 | 18-GR10-6 | BOLD:ADD1232 | C3 | UCOIF+UCOIR |
| GRBAL106-16 | 18-GR10-7 | BOLD:ADD1232 | C4 | UCOIF+UCOIR |
| GRBAL110-16 | 18-GR10-8 | BOLD:ADD1232 | C20 | UCOIF+UCOIR |
| GRBAL108-16 | 18-GR10-9 | BOLD:ADD1232 | C17 | UCOIF+UCOIR |
| GRBAL114-16 | 19-SB- BG29-1 | BOLD:ACQ3482 | C12 | UCOIF+UCOIR |
| GRBAL119-16 | 19-SB- BG29-10 | BOLD:ACQ3482 | C13 | UCOIF+UCOIR |
| GRBAL120-16 | 19-SB- BG29-11 | BOLD:ACQ3482 | C13 | UCOIF+UCOIR |
| GRBAL121-16 | 19-SB- BG29-12 | BOLD:ACQ3482 | C13 | UCOIF+UCOIR |
| GRBAL122-16 | 19-SB- BG29-2 | BOLD:ADD0637 | C16 | UCOIF+UCOIR |
| GRBAL115-16 | 19-SB- BG29-3 | BOLD:ACQ3482 | C13 | UCOIF+UCOIR |
| GRBAL112-16 | 19-SB- BG29-4 | BOLD:ACQ3482 | C10 | UCOIF+UCOIR |
| GRBAL116-16 | 19-SB- BG29-5 | BOLD:ACQ3482 | C13 | UCOIF+UCOIR |
| GRBAL113-16 | 19-SB- BG29-6 | BOLD:ACQ3482 | C11 | UCOIF+UCOIR |
| GRBAL117-16 | 19-SB- BG29-8 | BOLD:ACQ3482 | C13 | UCOIF+UCOIR |
| GRBAL118-16 | 19-SB- BG29-9 | BOLD:ACQ3482 | C13 | UCOIF+UCOIR |
| GRBAL001-16 | 1-GSSL-SLODRA-11 | BOLD:AAY1309 | C28 | LCO1490+HCO2198 |
| GRBAL002-16 | 1-GSSL-SLODRA-12 | BOLD:AAY1309 | C28 | LCO1490+HCO2198 |
| GRBAL003-16 | 1-GSSL-SLODRA-13 | BOLD:AAY1309 | C28 | LCO1490+HCO2198 |
| GRBAL125-16 | 20-GR38-15 | BOLD:ADD0637 | C15 | UCOIF+UCOIR |
| GRBAL123-16 | 20-GR38-3 | BOLD:ACQ3482 | C13 | UCOIF+UCOIR |
| GRBAL124-16 | 20-GR38-5 | BOLD:ACQ3482 | C13 | LCO1490+HCO2198 |
| GRBAL126-16 | 20-GR38-7 | BOLD:ADD2306 | C46 | LCO1490+HCO2198 |
| GRBAL127-16 | 20-GR38-8 | BOLD:ADD2306 | C47 | LCO1490+HCO2198 |
| GRBAL128-16 | 20-GR38-9 | BOLD:ADD2306 | C46 | LCO1490+HCO2198 |
| GRBAL129-16 | 21-GR07-1 | BOLD:ACH5400 | C5 | LCO1490+HCO2198 |
| GRBAL132-16 | 21-GR07-11 | BOLD:ACH5400 | C6 | UCOIF+UCOIR |
| GRBAL133-16 | 21-GR07-12 | BOLD:ACH5400 | C6 | UCOIF+UCOIR |
| GRBAL130-16 | 21-GR07-2 | BOLD:ACH5400 | C6 | LCO1490+HCO2198 |
| GRBAL131-16 | 21-GR07-5 | BOLD:ACH5400 | C6 | LCO1490+HCO2198 |
| GRBAL134-16 | 22-GR11-1 | BOLD:ACH5400 | C6 | UCOIF+UCOIR |
| GRBAL135-16 | 22-GR11-3 | BOLD:ACH5400 | C6 | UCOIF+UCOIR |
| GRBAL136-16 | 22-GR11-4 | BOLD:ACH5400 | C6 | UCOIF+UCOIR |
| GRBAL137-16 | 22-GR11-5 | BOLD:ACH5400 | C6 | UCOIF+UCOIR |
| GRBAL138-16 | 22-GR11-8 | BOLD:ACH5400 | C6 | LCO1490+HCO2198 |
| GRBAL142-16 | 23-GR33-1 | BOLD:ADD3864 | C56 | LCO1490+HCO2198 |
| GRBAL149-16 | 23-GR33-10 | BOLD:ADD3864 | C56 | UCOIF+UCOIR |
| GRBAL141-16 | 23-GR33-11 | BOLD:ADD3864 | C55 | LCO1490+HCO2198 |
| GRBAL140-16 | 23-GR33-12 | BOLD:ADD3864 | C54 | LCO1490+HCO2198 |
| GRBAL143-16 | 23-GR33-2 | BOLD:ADD3864 | C56 | LCO1490+HCO2198 |
| GRBAL144-16 | 23-GR33-3 | BOLD:ADD3864 | C56 | LCO1490+HCO2198 |
| GRBAL139-16 | 23-GR33-4 | BOLD:ADD3864 | C53 | LCO1490+HCO2198 |
| GRBAL145-16 | 23-GR33-5 | BOLD:ADD3864 | C56 | UCOIF+UCOIR |
| GRBAL146-16 | 23-GR33-6 | BOLD:ADD3864 | C56 | UCOIF+UCOIR |
| GRBAL147-16 | 23-GR33-7 | BOLD:ADD3864 | C56 | LCO1490+HCO2198 |
| GRBAL148-16 | 23-GR33-9 | BOLD:ADD3864 | C56 | LCO1490+HCO2198 |
| GRBAL154-16 | 24-GR24-1 | BOLD:ADD1703 | C76 | LCO1490+HCO2198 |
| GRBAL152-16 | 24-GR24-10 | BOLD:ADD1703 | C74 | LCO1490+HCO2198 |
| GRBAL153-16 | 24-GR24-11 | BOLD:ADD1703 | C74 | LCO1490+HCO2198 |
| GRBAL158-16 | 24-GR24-12 | BOLD:ADD1703 | C76 | LCO1490+HCO2198 |
| GRBAL155-16 | 24-GR24-2 | BOLD:ADD1703 | C76 | LCO1490+HCO2198 |
| GRBAL156-16 | 24-GR24-5 | BOLD:ADD1703 | C76 | LCO1490+HCO2198 |
| GRBAL150-16 | 24-GR24-7 | BOLD:ADD1703 | C74 | LCO1490+HCO2198 |
| GRBAL151-16 | 24-GR24-8 | BOLD:ADD1703 | C74 | LCO1490+HCO2198 |
| GRBAL157-16 | 24-GR24-9 | BOLD:ADD1703 | C76 | LCO1490+HCO2198 |
| GRBAL159-16 | 25-GR30-1 | BOLD:ADD3860 | C78 | LCO1490+HCO2198 |
| GRBAL167-16 | 25-GR30-12 | BOLD:ADD1702 | C83 | LCO1490+HCO2198 |
| GRBAL160-16 | 25-GR30-2 | BOLD:ADD3860 | C79 | UCOIF+UCOIR |
| GRBAL161-16 | 25-GR30-3 | BOLD:ADD1702 | C80 | LCO1490+HCO2198 |
| GRBAL162-16 | 25-GR30-5 | BOLD:ADD1702 | C80 | LCO1490+HCO2198 |
| GRBAL163-16 | 25-GR30-6 | BOLD:ADD1702 | C80 | LCO1490+HCO2198 |
| GRBAL164-16 | 25-GR30-7 | BOLD:ADD1702 | C80 | LCO1490+HCO2198 |
| GRBAL165-16 | 25-GR30-8 | BOLD:ADD1702 | C80 | LCO1490+HCO2198 |
| GRBAL166-16 | 25-GR30-9 | BOLD:ADD1702 | C83 | LCO1490+HCO2198 |
| GRBAL168-16 | 26-GR25-1 | BOLD:ADD1703 | C72 | LCO1490+HCO2198 |
| GRBAL174-16 | 26-GR25-10 | BOLD:ADD1703 | C72 | LCO1490+HCO2198 |
| GRBAL175-16 | 26-GR25-11 | BOLD:ADD1703 | C72 | LCO1490+HCO2198 |
| GRBAL176-16 | 26-GR25-12 | BOLD:ADD1703 | C73 | LCO1490+HCO2198 |
| GRBAL169-16 | 26-GR25-2 | BOLD:ADD1703 | C72 | LCO1490+HCO2198 |
| GRBAL170-16 | 26-GR25-3 | BOLD:ADD1703 | C72 | UCOIF+UCOIR |
| GRBAL171-16 | 26-GR25-4 | BOLD:ADD1703 | C72 | UCOIF+UCOIR |
| GRBAL172-16 | 26-GR25-7 | BOLD:ADD1703 | C72 | LCO1490+HCO2198 |
| GRBAL173-16 | 26-GR25-9 | BOLD:ADD1703 | C72 | LCO1490+HCO2198 |
| GRBAL005-16 | 2-GSSLO-SLONVD-3 | BOLD:AAY1309 | C28 | LCO1490+HCO2198 |
| GRBAL004-16 | 2-GSSLO-SLONVD-4 | BOLD:ADD4052 | C25 | LCO1490+HCO2198 |
| GRBAL006-16 | 3-GSHR-(C)HRVAR-8 | BOLD:ACZ9504 | C23 | LCO1490+HCO2198 |
| GRBAL013-16 | 4-MR- BG01-12 | BOLD:ACZ9504 | C24 | UCOIF+UCOIR |
| GRBAL007-16 | 4-MR-BG01-1 | BOLD:ACZ9504 | C24 | LCO1490+HCO2198 |
| GRBAL008-16 | 4-MR-BG01-2 | BOLD:ACZ9504 | C24 | LCO1490+HCO2198 |
| GRBAL009-16 | 4-MR-BG01-4 | BOLD:ACZ9504 | C24 | LCO1490+HCO2198 |
| GRBAL010-16 | 4-MR-BG01-6 | BOLD:ACZ9504 | C24 | LCO1490+HCO2198 |
| GRBAL011-16 | 4-MR-BG01-7 | BOLD:ACZ9504 | C24 | UCOIF+UCOIR |
| GRBAL012-16 | 4-MR-BG01-9 | BOLD:ACZ9504 | C24 | UCOIF+UCOIR |
| GRBAL014-16 | 5-GSSRB-SRBPRE-6 | BOLD:ACI2262 | C52 | LCO1490+HCO2198 |
| GRBAL016-16 | 6-AL18-DRI-1 | BOLD:ACH5401 | C44 | UCOIF+UCOIR |
| GRBAL017-16 | 6-AL18-DRI-2 | BOLD:ACH5401 | C44 | UCOIF+UCOIR |
| GRBAL018-16 | 6-AL18-DRI-3 | BOLD:ACH5401 | C44 | COIGrF+COIGrR2 |
| GRBAL015-16 | 6-AL18-DRI-4 | BOLD:ACH5401 | C32 | UCOIF+UCOIR |
| GRBAL019-16 | 6-AL18-DRI-5 | BOLD:ADD4054 | C45 | COIGrF+COIGrR2 |
| GRBAL020-16 | 6-AL18-DRI-8 | BOLD:ADD4054 | C45 | COIGrF+COIGrR2 |
| GRBAL023-16 | 7-AL36-OHR-1R | BOLD:ACH5401 | C32 | UCOIF+UCOIR |
| GRBAL024-16 | 7-AL36-OHR-2R | BOLD:ACH5401 | C32 | UCOIF+UCOIR |
| GRBAL025-16 | 7-AL36-OHR-3R | BOLD:ACH5401 | C32 | UCOIF+UCOIR |
| GRBAL022-16 | 7-AL36-OHR-5 | BOLD:ACH5401 | C32 | UCOIF+UCOIR |
| GRBAL021-16 | 7-AL36-OHR-6 | BOLD:ADD4053 | C31 | UCOIF+UCOIR |
| GRBAL028-16 | 8-PRE-AL44-1 | BOLD:ACZ6711 | C40 | UCOIF+UCOIR |
| GRBAL029-16 | 8-PRE-AL44-2 | BOLD:ACZ6711 | C41 | UCOIF+UCOIR |
| GRBAL026-16 | 8-PRE-AL44-3 | BOLD:ACZ6711 | C34 | UCOIF+UCOIR |
| GRBAL027-16 | 8-PRE-AL44-6 | BOLD:ACZ6711 | C35 | UCOIF+UCOIR |
| GRBAL033-16 | 9-GR04-1 | BOLD:ACZ6711 | C38 | UCOIF+UCOIR |
| GRBAL038-16 | 9-GR04-10 | BOLD:ACZ6711 | C38 | UCOIF+UCOIR |
| GRBAL030-16 | 9-GR04-2 | BOLD:ACZ6711 | C35 | UCOIF+UCOIR |
| GRBAL031-16 | 9-GR04-3 | BOLD:ACZ6711 | C35 | UCOIF+UCOIR |
| GRBAL032-16 | 9-GR04-4 | BOLD:ACZ6711 | C35 | UCOIF+UCOIR |
| GRBAL039-16 | 9-GR04-5 | BOLD:ACZ6711 | C42 | UCOIF+UCOIR |
| GRBAL034-16 | 9-GR04-6 | BOLD:ACZ6711 | C38 | UCOIF+UCOIR |
| GRBAL035-16 | 9-GR04-7 | BOLD:ACZ6711 | C38 | UCOIF+UCOIR |
| GRBAL036-16 | 9-GR04-8 | BOLD:ACZ6711 | C38 | UCOIF+UCOIR |
| GRBAL037-16 | 9-GR04-9 | BOLD:ACZ6711 | C38 | UCOIF+UCOIR |
