# Supplementary material for: Neogene paleogeography provides context for understanding the origin and spatial distribution of cryptic diversity in a widespread Balkan freshwater amphipod
Source: PeerJ. 2017 Feb 28;5:e3016. doi: 10.7717/peerj.3016 (PMC5333542; doi:10.7717/peerj.3016)
Supplement: Table S2 — Divergence time (Ma) for key nodes and rates of COI evolution in substitutions per site, per My estimated using Bayesian inference for different calibration schemes. Node affiliation given in Fig. S1. Calibration schemes based on secondary calibration points, standard rate and geological points (see material and methods). Lower and upper 95% highest posterior densities (95%HPD) are provided. [file peerj-05-3016-s002.docx]

|  | Secondary | | Rate | | Geology | |
| --- | --- | --- | --- | --- | --- | --- |
| Node | Median | 95%HPD | Median | 95%HPD | Median | 95%HPD |
| All | 17.75 | 14.38-21.12 | 17.52 | 14.24-21.48 | 16.15 | 9.97-24.36 |
| A-H | 12.4 | 9.52-15.48 | 11.92 | 9.33-15.05 | 10.82 | 6.33-16.58 |
| A-G | 7.89 | 5.96-9.84 | 7.66 | 6.08-9.49 | 7.09 | 4.19-10.74 |
| A-E | 6.67 | 5.05-8.47 | 6.41 | 5.03-8.01 | 5.93 | 3.49-9.08 |
| A-C | 4.47 | 3.24-5.78 | 4.3 | 3.24-5.46 | 3.99 | 2.45-6.33 |
| AB | 3.8 | 2.66-4.99 | 3.64 | 2.61-4.7 | 3.43 | 1.97-5.41 |
| DE | 3.65 | 2.41-5.06 | 3.53 | 2.38-4.07 | 3.3 | 1.82-5.43 |
| FG | 5.77 | 4-7.69 | 5.57 | 3.98-7.34 | 5.04 | 2.85-8.04 |
| I-M | 13.2 | 9.99-16.22 | 13.02 | 10.27-16.34 | 12.18 | 7.12-18.47 |
| IJ | 10.11 | 7.17-12.97 | 9.94 | 7.29-12.86 | 9.37 | 5.37-14.54 |
| K-M | 7.39 | 5.28-9.67 | 7.27 | 5.33-9.51 | 6.68 | 3.58-10.59 |
| LM | 3.3 | 2.16-4.52 | 3.22 | 2.21-4.42 | 2.91 | 1.54-4.71 |
| Rate: | 0.0113 | 0.0083-0.0144 | 0.0115 | NA | 0.0130 | 0.0077-0.0193 |
