# Supplementary material for: Neogene paleogeography provides context for understanding the origin and spatial distribution of cryptic diversity in a widespread Balkan freshwater amphipod
Source: PeerJ. 2017 Feb 28;5:e3016. doi: 10.7717/peerj.3016 (PMC5333542; doi:10.7717/peerj.3016)
Supplement: Table S3 — Node ages (Ma) with lower and upper 95% highest posterior densities, geographical coordinates of the ancestral locations expressed as mean and standard deviation (SD), probabilities associated with occurrence in riverine (P(R)) or in lacustrine (P(L)) habitat (see Fig. S1 for node identification). [file peerj-05-3016-s003.docx]

| Node ID |  | Node Age |  |  |  | Geography |  |  |  |  |  | Habitat |  |
| --- | --- | --- | --- | --- | --- | --- | --- | --- | --- | --- | --- | --- | --- |
|  |  | Value | Lower 95% HPD | Upper 95%  HPD |  | Latitude mean | SD |  | Longitude mean | SD |  | P(River) | P(Lake) |
| Full |  | 17.75 | 14.38 | 21.12 |  | 41.6163 | 0.6216 |  | 20.6677 | 1.7187 |  | 0.51 | 0.49 |
| A-H |  | 12.4 | 9.52 | 15.48 |  | 41.6163 | 0.6216 |  | 20.6677 | 1.7187 |  | 0.51 | 0.49 |
| A-G |  | 7.89 | 5.96 | 9.84 |  | 41.6163 | 0.6216 |  | 20.6677 | 1.7187 |  | 0.51 | 0.49 |
| A-E |  | 6.67 | 5.05 | 8.47 |  | 42.0154 | 0.5130 |  | 20.5030 | 1.3387 |  | 0.58 | 0.42 |
| A-C |  | 4.47 | 3.24 | 5.78 |  | 42.8904 | 0.4739 |  | 20.1473 | 1.2876 |  | 0.56 | 0.44 |
| AB |  | 3.80 | 2.66 | 4.99 |  | 42.5658 | 0.5000 |  | 20.4741 | 1.3257 |  | 0.48 | 0.52 |
| A1 |  | 1.24 | 0.78 | 1.73 |  | 41.2711 | 0.2686 |  | 21.8294 | 0.6876 |  | 0.29 | 0.71 |
| A2 |  | 1.04 | 0.64 | 1.47 |  | 41.2182 | 0.2426 |  | 22.0563 | 0.6485 |  | 0.24 | 0.76 |
| A3 |  | 0.91 | 0.52 | 1.34 |  | 41.1845 | 0.2594 |  | 21.6798 | 0.6706 |  | 0.53 | 0.47 |
| A4 |  | 0.74 | 0.30 | 1.11 |  | 41.2669 | 0.2182 |  | 22.7765 | 0.5829 |  | 0.96 | 0.04 |
| A5 |  | 0.59 | 0.27 | 0.95 |  | 40.8672 | 0.1937 |  | 21.8666 | 0.5028 |  | 0.00 | 1.00 |
| A6 |  | 0.39 | 0.14 | 0.68 |  | 40.7799 | 0.1546 |  | 21.7918 | 0.4166 |  | 0.00 | 1.00 |
| A7 |  | 0.32 | 0.11 | 0.57 |  | 41.3639 | 0.1538 |  | 23.1445 | 0.4271 |  | 0.99 | 0.01 |
| A8 |  | 0.28 | 0.05 | 0.56 |  | 40.5410 | 0.1690 |  | 21.4176 | 0.4433 |  | 0.20 | 0.80 |
| A9 |  | 0.23 | 0.04 | 0.45 |  | 41.3182 | 0.1512 |  | 23.2632 | 0.3936 |  | 0.99 | 0.01 |
| A10 |  | 0.19 | 0.03 | 0.39 |  | 41.3567 | 0.1257 |  | 23.2521 | 0.3302 |  | 0.99 | 0.01 |
| A11 |  | 0.19 | 0.01 | 0.41 |  | 40.7501 | 0.1315 |  | 21.7669 | 0.3418 |  | 0.00 | 1.00 |
| A12 |  | 0.20 | 0.03 | 0.4 |  | 40.7599 | 0.1274 |  | 21.6748 | 0.3283 |  | 0.00 | 1.00 |
| A13 |  | 0.17 | 0.03 | 0.34 |  | 40.7502 | 0.1134 |  | 21.7928 | 0.2972 |  | 0.00 | 1.00 |
| A14 |  | 0.10 | 0.00 | 0.23 |  | 41.4068 | 0.0989 |  | 23.2867 | 0.2589 |  | 1.00 | 0.00 |
| A15 |  | 0.09 | 0.00 | 0.22 |  | 40.7495 | 0.0941 |  | 21.7517 | 0.2477 |  | 0.00 | 1.00 |
| A16 |  | 0.08 | 0.00 | 0.20 |  | 40.7309 | 0.0905 |  | 21.6991 | 0.2406 |  | 0.00 | 1.00 |
| C1 |  | 1.68 | 0.93 | 2.50 |  | 45.5519 | 0.3766 |  | 17.3559 | 1.0065 |  | 0.84 | 0.16 |
| C2 |  | 0.83 | 0.35 | 1.38 |  | 45.8312 | 0.2694 |  | 17.5408 | 0.7257 |  | 0.93 | 0.07 |
| C3 |  | 0.15 | 0.00 | 0.37 |  | 45.6510 | 0.1292 |  | 18.8627 | 0.3449 |  | 1.00 | 0.00 |
| DE |  | 3.65 | 2.41 | 5.06 |  | 41.5679 | 0.5189 |  | 21.0736 | 1.3386 |  | 0.70 | 0.30 |
| E1 |  | 2.07 | 1.30 | 2.87 |  | 41.1126 | 0.3652 |  | 21.3797 | 0.9788 |  | 0.85 | 0.15 |
| E2 |  | 1.62 | 1.01 | 2.27 |  | 41.0342 | 0.3247 |  | 21.5164 | 0.8531 |  | 0.88 | 0.12 |
| E3 |  | 0.98 | 0.57 | 1.43 |  | 40.8270 | 0.2392 |  | 21.0791 | 0.6626 |  | 0.95 | 0.05 |
| E4 |  | 0.84 | 0.46 | 1.25 |  | 40.8141 | 0.2423 |  | 20.9617 | 0.6387 |  | 0.91 | 0.09 |
| E5 |  | 0.21 | 0.00 | 0.50 |  | 40.7216 | 0.1465 |  | 20.9067 | 0.3850 |  | 0.99 | 0.01 |
| FG |  | 5.77 | 4.00 | 7.69 |  | 41.2323 | 0.6063 |  | 21.1947 | 1.5681 |  | 0.42 | 0.58 |
| F1 |  | 0.42 | 0.15 | 0.74 |  | 40.5960 | 0.1885 |  | 22.4606 | 0.4918 |  | 0.99 | 0.01 |
| F2 |  | 0.27 | 0.08 | 0.51 |  | 40.5852 | 0.1472 |  | 22.4757 | 0.3844 |  | 1.00 | 0.00 |
| F3 |  | 0.19 | 0.02 | 0.41 |  | 40.5892 | 0.1266 |  | 22.4445 | 0.3324 |  | 0.99 | 0.01 |
| G1 |  | 1.52 | 0.90 | 2.23 |  | 41.0251 | 0.3168 |  | 20.9191 | 0.8561 |  | 0.10 | 0.90 |
| G2 |  | 0.81 | 0.43 | 1.23 |  | 41.1973 | 0.2389 |  | 20.5478 | 0.6222 |  | 0.50 | 0.50 |
| G3 |  | 0.66 | 0.34 | 1.01 |  | 41.2673 | 0.1920 |  | 20.4036 | 0.4990 |  | 0.97 | 0.03 |
| G4 |  | 0.52 | 0.21 | 0.88 |  | 40.9010 | 0.2017 |  | 20.9271 | 0.5237 |  | 0.00 | 1.00 |
| G5 |  | 0.43 | 0.14 | 0.76 |  | 41.4382 | 0.1725 |  | 20.2374 | 0.4436 |  | 0.91 | 0.09 |
| G6 |  | 0.37 | 0.14 | 0.64 |  | 40.8703 | 0.1519 |  | 20.9662 | 0.3983 |  | 0.00 | 1.00 |
| G7 |  | 0.35 | 0.12 | 0.62 |  | 41.1536 | 0.1603 |  | 20.4077 | 0.4296 |  | 0.99 | 0.01 |
| G8 |  | 0.24 | 0.05 | 0.47 |  | 40.8241 | 0.1291 |  | 21.0518 | 0.3356 |  | 0.00 | 1.00 |
| G9 |  | 0.22 | 0.03 | 0.43 |  | 41.1044 | 0.1347 |  | 20.4751 | 0.3566 |  | 0.99 | 0.01 |
| G10 |  | 0.20 | 0.01 | 0.47 |  | 41.8548 | 0.1342 |  | 19.6963 | 0.3537 |  | 0.99 | 0.01 |
| G11 |  | 0.17 | 0.02 | 0.36 |  | 40.8595 | 0.1290 |  | 20.9572 | 0.3385 |  | 0.00 | 1.00 |
| G12 |  | 0.15 | 0.00 | 0.33 |  | 40.8441 | 0.1139 |  | 21.0150 | 0.2981 |  | 0.00 | 1.00 |
| G13 |  | 0.10 | 0.00 | 0.25 |  | 40.9169 | 0.1043 |  | 20.9216 | 0.2723 |  | 0.00 | 1.00 |
| H1 |  | 0.48 | 0.18 | 0.84 |  | 40.3154 | 0.1992 |  | 19.5575 | 0.5314 |  | 0.98 | 0.02 |
| H2 |  | 0.29 | 0.08 | 0.53 |  | 40.3271 | 0.1520 |  | 19.5098 | 0.3992 |  | 0.99 | 0.01 |
| H3 |  | 0.15 | 0.02 | 0.32 |  | 40.3378 | 0.1168 |  | 19.4687 | 0.3068 |  | 1.00 | 0.00 |
| I-M |  | 13.2 | 9.99 | 16.22 |  | 39.9865 | 0.7445 |  | 21.2102 | 1.9038 |  | 0.50 | 0.50 |
| IJ |  | 10.11 | 7.17 | 12.97 |  | 39.8772 | 0.8552 |  | 21.1711 | 2.1621 |  | 0.48 | 0.52 |
| I1 |  | 0.52 | 0.22 | 0.86 |  | 38.6255 | 0.2156 |  | 21.5542 | 0.5559 |  | 0.00 | 1.00 |
| I2 |  | 0.36 | 0.13 | 0.63 |  | 38.6186 | 0.1657 |  | 21.5149 | 0.4292 |  | 0.00 | 1.00 |
| I3 |  | 0.24 | 0.04 | 0.47 |  | 38.6086 | 0.1449 |  | 21.5133 | 0.3818 |  | 0.00 | 1.00 |
| I4 |  | 0.17 | 0.03 | 0.34 |  | 38.5929 | 0.1196 |  | 21.5585 | 0.3036 |  | 0.00 | 1.00 |
| I5 |  | 0.09 | 0.00 | 0.22 |  | 38.5961 | 0.0953 |  | 21.5400 | 0.2501 |  | 0.00 | 1.00 |
| J1 |  | 0.15 | 0.00 | 0.36 |  | 40.7030 | 0.1315 |  | 20.8692 | 0.3443 |  | 1.00 | 0.00 |
| K-M |  | 7.39 | 5.28 | 9.67 |  | 39.6228 | 0.7943 |  | 21.7679 | 2.1187 |  | 0.55 | 0.45 |
| K1 |  | 0.34 | 0.09 | 0.62 |  | 39.0812 | 0.1601 |  | 22.0773 | 0.4206 |  | 0.99 | 0.01 |
| K2 |  | 0.15 | 0.01 | 0.33 |  | 39.1478 | 0.1220 |  | 22.0593 | 0.3236 |  | 1.00 | 0.00 |
| K3 |  | 0.12 | 0.00 | 0.29 |  | 38.9698 | 0.1113 |  | 22.2005 | 0.2930 |  | 1.00 | 0.00 |
| LM |  | 3.3 | 2.16 | 4.52 |  | 39.7913 | 0.5288 |  | 21.6471 | 1.3596 |  | 0.71 | 0.29 |
| L1 |  | 1.49 | 0.84 | 2.21 |  | 39.8434 | 0.3487 |  | 22.3361 | 0.8617 |  | 0.87 | 0.13 |
| L2 |  | 0.61 | 0.22 | 1.05 |  | 39.8718 | 0.2395 |  | 22.5165 | 0.6440 |  | 0.95 | 0.05 |
| L3 |  | 0.21 | 0.02 | 0.46 |  | 39.8744 | 0.1494 |  | 22.5674 | 0.3920 |  | 0.99 | 0.01 |
| M1 |  | 0.49 | 0.2 | 0.83 |  | 39.7399 | 0.1905 |  | 20.5976 | 0.5124 |  | 0.99 | 0.01 |
| M2 |  | 0.25 | 0.03 | 0.45 |  | 39.7324 | 0.1269 |  | 20.5680 | 0.3331 |  | 1.00 | 0.00 |
| M3 |  | 0.28 | 0.05 | 0.55 |  | 39.7225 | 0.1576 |  | 20.6448 | 0.4105 |  | 0.99 | 0.01 |
| M4 |  | 0.20 | 0.08 | 0.45 |  | 39.7145 | 0.1086 |  | 20.6173 | 0.2850 |  | 1.00 | 0.00 |
| M5 |  | 0.16 | 0.03 | 0.31 |  | 39.7237 | 0.1002 |  | 20.5972 | 0.2659 |  | 1.00 | 0.00 |
| M6 |  | 0.10 | 0.00 | 0.24 |  | 39.7132 | 0.0910 |  | 20.6300 | 0.2408 |  | 1.00 | 0.00 |
