# Supplementary material for: Neogene paleogeography provides context for understanding the origin and spatial distribution of cryptic diversity in a widespread Balkan freshwater amphipod
Source: PeerJ. 2017 Feb 28;5:e3016. doi: 10.7717/peerj.3016 (PMC5333542; doi:10.7717/peerj.3016)
Supplement: Table S4 — Mean Kimura two parameters (K2p) distances (below the diagonal) and standard error (SE) (above the diagonal) between MOTUs for 74 COI haplotypes. N and k = number of individual sampled and haplotypes per MOTU, respectively. [file peerj-05-3016-s004.docx]

| MOTUs |  |  |  |  | Bet | ween | MOTUs |  |  |  |  |  |  |  |  |  |  |
| --- | --- | --- | --- | --- | --- | --- | --- | --- | --- | --- | --- | --- | --- | --- | --- | --- | --- |
|  |  | N | k |  | A | B | C | D | E | F | G | H | I | J | K | L | M |
| **A** |  | 49 | *18* |  |  | 0.012 | 0.010 | 0.011 | 0.015 | 0.017 | 0.015 | 0.019 | 0.021 | 0.020 | 0.023 | 0.022 | 0.022 |
| **B** |  | 1 | 1 |  | 0.087 |  | 0.012 | 0.014 | 0.016 | 0.017 | 0.016 | 0.020 | 0.021 | 0.022 | 0.024 | 0.023 | 0.023 |
| **C** |  | 13 | 4 |  | 0.073 | 0.080 |  | 0.012 | 0.014 | 0.017 | 0.014 | 0.020 | 0.022 | 0.023 | 0.025 | 0.023 | 0.022 |
| **D** |  | 2 | 1 |  | 0.080 | 0.098 | 0.077 |  | 0.010 | 0.015 | 0.013 | 0.018 | 0.022 | 0.021 | 0.022 | 0.023 | 0.021 |
| **E** |  | 13 | 6 |  | 0.132 | 0.124 | 0.113 | 0.060 |  | 0.014 | 0.014 | 0.018 | 0.021 | 0.021 | 0.022 | 0.022 | 0.020 |
| **F** |  | 11 | 4 |  | 0.140 | 0.132 | 0.134 | 0.106 | 0.110 |  | 0.013 | 0.019 | 0.021 | 0.024 | 0.021 | 0.021 | 0.021 |
| **G** |  | 40 | 14 |  | 0.126 | 0.124 | 0.105 | 0.095 | 0.114 | 0.101 |  | 0.019 | 0.020 | 0.021 | 0.021 | 0.020 | 0.019 |
| **H** |  | 4 | 4 |  | 0.180 | 0.178 | 0.188 | 0.151 | 0.168 | 0.166 | 0.172 |  | 0.024 | 0.022 | 0.022 | 0.022 | 0.022 |
| **I** |  | 12 | 6 |  | 0.193 | 0.177 | 0.199 | 0.188 | 0.187 | 0.174 | 0.169 | 0.224 |  | 0.019 | 0.02 | 0.020 | 0.020 |
| **J** |  | 4 | 2 |  | 0.181 | 0.184 | 0.203 | 0.176 | 0.194 | 0.221 | 0.188 | 0.207 | 0.154 |  | 0.019 | 0.019 | 0.018 |
| **K** |  | 18 | 4 |  | 0.220 | 0.207 | 0.226 | 0.202 | 0.211 | 0.190 | 0.191 | 0.218 | 0.196 | 0.166 |  | 0.015 | 0.016 |
| **L** |  | 9 | 4 |  | 0.217 | 0.222 | 0.225 | 0.212 | 0.214 | 0.190 | 0.196 | 0.214 | 0.184 | 0.171 | 0.119 |  | 0.010 |
| **M** |  | 10 | 7 |  | 0.218 | 0.213 | 0.205 | 0.192 | 0.194 | 0.184 | 0.179 | 0.211 | 0.176 | 0.154 | 0.122 | 0.060 |  |
