# Supplementary material for: Neogene paleogeography provides context for understanding the origin and spatial distribution of cryptic diversity in a widespread Balkan freshwater amphipod
Source: PeerJ. 2017 Feb 28;5:e3016. doi: 10.7717/peerj.3016 (PMC5333542; doi:10.7717/peerj.3016)
Supplement: Table S5 — Fit of the diversification models to Bayesian reconstruction of phylogeny, based on the Akaike information criterion (AIC). [file peerj-05-3016-s005.docx]

| Model | Pure birth | DDL | DDX | Birth death | Yule2rate | Yule3rate |
| --- | --- | --- | --- | --- | --- | --- |
| AIC | 37.31389 | 37.52576 | 38.66991 | 39.31389 | 39.85968 | 40.53367 |

Pure birth – constant rate, no extinction

Birth death – speciation and extinction under constant rate

DDL/DDX: density dependent speciation models

Yule x-rate models – Yule model of speciation with x as the number of different rates observed along the speciation
