# Supplementary material for: Neogene paleogeography provides context for understanding the origin and spatial distribution of cryptic diversity in a widespread Balkan freshwater amphipod
Source: PeerJ. 2017 Feb 28;5:e3016. doi: 10.7717/peerj.3016 (PMC5333542; doi:10.7717/peerj.3016)
Supplement: Table S6 — Molecular genetic diversity and historical demography based on mtDNA COI gene region (530 bp) within the 13 MOTUs (A–M). Analysis was done either at the scale of the entire MOTU (in bold) or a site or set of sites (in italic) within a MOTU. [file peerj-05-3016-s006.docx]

| MOTUs (sites) | N | **k** | **nps** | **H +/- SD** | Π | **D** | **p^a^** | **Fs** | **p^a^** |
| --- | --- | --- | --- | --- | --- | --- | --- | --- | --- |
| **A (all)** | **49** | ***18*** | **37** | **0.90 +/- 0.02** | **0.0153 +/- 0.0080** | **-0.078** | **0.51** | **-0.694** | **0.44** |
| *A (19)* | *11* | *5* | *12* | *0.62 +/- 0.16* | *0.0044 +/- 0.00293* | *-1.876* | *0.01* | *-0.043* | *0.47* |
| *A (16)* | *7* | *1* | *0* | *0* | *0* | *N.A* | *N.A* | *N.A* | *N.A* |
| *A (21+22)* | *10* | *2* | *3* | *0.20 +/- 0.15* | *0.0011 +/- 0.0011* | *-1.562* | *0.049* | *1.224* | *0.68* |
| *A (17)* | *9* | *3* | *2* | *0.55 +/- 0.16* | *0.0011 +/- 0.0011* | *-0.583* | *0.29* | *-0.532* | *0.12* |
| *A (18)* | *9* | *6* | *6* | *0.89 +/- 0.09* | *0.0044 +/- 0.0030* | *0.248* | *0.66* | *-1.776* | *N.A.* |
| *A (20)* | *3* | *2* | *7* | *0.67 +/- 0.31* | *0.0088 +/- 0.0073* | *-0.465* | *0.74* | *2.884* | *0.86* |
| **B** | **1** | **1** | ***0*** | **0** | **0** | **0** | **1.0** | **N.A** | **N.A** |
| **C (all)** | **13** | **4** | **21** | **0.65 +/- 0.11** | **0.0151 +/- 0.0084** | **0.782** | **0.81** | **6.093** | **0.99** |
| *C (1+2+3)* | *6* | *4* | *21* | 0.80 +/- 0.17 | 0.0177 +/- 0.0109 | 0.138 | 0.56 | 2.448 | 0.85 |
| *C (4)* | *7* | *1* | *0* | *0* | *0* | *N.A* | *N.A* | *N.A* | *N.A* |
| **D** | **2** | **1** | **0** | **0** | **0** | **0** | **1.0** | **N.A** | **N.A** |
| **E (all)** | **13** | **6** | **40** | **0.83 +/- 0.07** | **0.0189 +/- 0.0104** | **-0.983** | **0.17** | **3.621** | **0.94** |
| *E (11+13)* | *9* | *5* | *37* | 0.80 +/- 0.12 | 0.0173 +/- 0.0100 | -1.641 | 0.032 | 2.798 | 0.882 |
| *E (20)* | *4* | *1* | *1* | *0* | *0* | *N.A* | *N.A* | *N.A* | *N.A* |
| **F** | **11** | **4** | **7** | **0.49 +/- 0.17** | **0.0033 +/- 0.0023** | **-0.586** | **0.31** | **0.458** | **0.60** |
| **G (all)** | **40** | **14** | **26** | **0.82 +/- 0.05** | **0.0112 +/- 0.0060** | **-0.320** | **0.45** | **-0.563** | **0.45** |
| *G (6+7)* | **11** | 4 | 12 | 0.74 +/- 0.09 | 0.0066 +/- 0.0041 | -0.603 | 0.272 | 2.228 | 0.883 |
| *G (8+9+10)* | **26** | 7 | 6 | 0.61 +/- 0.10 | 0.0023 +/- 0.0017 | -0.654 | 0.275 | -2.279 | 0.058 |
| *G (11)* | **3** | 3 | 6 | 1.00 +/- 0.27 | 0.0075 +/- 0.0064 | 0.000 | 0.772 | 0.133 | 0.263 |
| **H** | **4** | **4** | **6** | **1.00 +/- 0.17** | **0.0066 +/- 0.0050** | **0.674** | **0.83** | **-1.012** | **0.12** |
| **I** | **12** | **6** | **8** | **0.68 +/- 0.14** | **0.0036 +/- 0.0025** | **-1.047** | **0.17** | **-1.390** | **0.15** |
| **J** | **4** | **2** | **1** | **0.50 +/- 0.26** | **0.0009 +/- 0.0011** | **-0.612** | **0.36** | **0.172** | **0.34** |
| **K (all)** | **18** | **4** | **4** | **0.71 +/- 0.06** | **0.0028 +/- 0.0020** | **0.945** | **0.83** | **0.889** | **0.73** |
| *K (24)* | *9* | *2* | 1 | 0.55 +/- 0.09 | 0.0010 +/- 0.0010 | -0.036 | 0.97 | 1.015 | 0.64 |
| *K (26)* | *9* | *2* | 2 | 0.22 +/- 0.16 | 0.0008 +/- 0.0009 | -1.362 | 0.09 | 0.671 | 0.45 |
| **L** | **9** | **4** | **21** | **0.69 +/- 0.14** | **0.0132 +/- 0.0077** | **-0.464** | **0.33** | **3.650** | **0.96** |
| **M** | **10** | **7** | **8** | **0.91 +/- 0.07** | **0.0036 +/- 0.0025** | **-1.399** | **0.09** | **-3.423*** | **0.003** |

1. Significance values (*p*) of the parameters were evaluated with 1000 simulations. D and Fu’s FS values represented with asterisk: *P < 0.05; **P < 0.01 (also **P < 0.05 after Bonferroni correction)
