# Supplementary material for: Neogene paleogeography provides context for understanding the origin and spatial distribution of cryptic diversity in a widespread Balkan freshwater amphipod
Source: PeerJ. 2017 Feb 28;5:e3016. doi: 10.7717/peerj.3016 (PMC5333542; doi:10.7717/peerj.3016)
Supplement: Table S7 — Genetic differentiation based on mtDNA COI gene region (530 bp) between sites (S1 vs S2) within MOTUs (A, C, E, G and K) present in more than one site within the morphospecies Gammarus roeselii in the Balkans. [file peerj-05-3016-s007.docx]

| M | S1 | S2 | N1/N2 | **F_ST_** | **ETSD** |  |
| --- | --- | --- | --- | --- | --- | --- |
| **A** | 19 | 16 | 11/7 | 0.635*** | <0.001*** |  |
|  | 19 | 21+22 | 11/10 | 0.583*** | <0.001*** |  |
|  | 19 | 17 | 11/9 | 0.411*** | <0.001*** |  |
|  | 19 | 18 | 11/9 | 0.252*** | <0.001*** |  |
|  | 19 | 20 | 11/3 | -0.106^ns^ | 0.764 ^ns^ |  |
|  | 16 | 21+22 | 7/10 | 0.881*** | <0.001*** |  |
|  | 16 | 17 | 7/9 | 0.691*** | <0.001*** |  |
|  | 16 | 18 | 7/9 | 0.515*** | <0.001*** |  |
|  | 16 | 20 | 7/3 | 0.815*** | 0.008** |  |
|  | 21+22 | 17 | 10/9 | 0.629*** | <0.001*** |  |
|  | 21+22 | 18 | 10/9 | 0.467*** | <0.001*** |  |
|  | 21+22 | 20 | 10/3 | 0.687*** | 0.007** |  |
|  | 17 | 18 | 9/9 | 0.277*** | <0.001*** |  |
|  | 17 | 20 | 9/3 | 0.410* | 0.013* |  |
|  | 18 | 20 | 9/3 | 0.187^ns^ | 0.096 ^ns^ |  |
| **C** | 1+2+3 | 4 | 7/6 | 0.618*** | <0.001*** |  |
| **E** | 11+13 | 20 | 9/4 | 0.520*** | 0.006** |  |
| **G** | 6+7 | 8+9+10 | 11/26 | 0.810*** | <0.001*** |  |
|  | 6+7 | 11 | 11/3 | 0.425** | 0.025* |  |
|  | 8+9+10 | 11 | 26/3 | 0.863*** | 0.009** |  |
| **K** | 24 | 26 | 9/9 | 0.795*** | <0.001*** |  |
